# Quantitative Trait Loci Mapping Identified Candidate Genes Involved in Plant Height Regulation in Rice

**DOI:** 10.3390/ijms242316895

**Published:** 2023-11-29

**Authors:** Jae-Ryoung Park, Yoon-Hee Jang, Eun-Gyeong Kim, Sang-Sun Hur, Kyung-Min Kim

**Affiliations:** 1 Crop Breeding Division, National Institute of Crop Science, Rural Development Administration, Wanju 55365, Republic of Korea; icd0192@korea.kr; 2 Coastal Agriculture Research Institute, Kyungpook National University, Daegu 41566, Republic of Korea; dkqkxk632@naver.com; 3 Division of Health and Welfare, Department of BioFood Science, Joongbu University, Geunmsan 32713, Republic of Korea; sshur@joongbu.ac.kr; 4 Department of Applied Biosciences, Kyungpook National University, Daegu 41566, Republic of Korea

**Keywords:** breeding, gibberellin, plant height, quantitative trait loci, rice

## Abstract

Rice plant height is an agricultural trait closely related to biomass, lodging tolerance, and yield. Identifying quantitative trait loci (QTL) regions related to plant height regulation and developing strategies to screen potential candidate genes can improve agricultural traits in rice. In this study, a double haploid population (CNDH), derived by crossing ‘Cheongcheong’ and ‘Nagdong’ individuals, was used, and a genetic map was constructed with 222 single-sequence repeat markers. In the RM3482-RM212 region on chromosome 1, *qPh1*, *qPh1-1*, *qPh1-3*, *qPh1-5*, and *qPh1-6* were identified for five consecutive years. The phenotypic variance explained ranged from 9.3% to 13.1%, and the LOD score ranged between 3.6 and 17.6. *OsPHq1*, a candidate gene related to plant height regulation, was screened in RM3482-RM212. *OsPHq1* is an ortholog of gibberellin 20 oxidase 2, and its haplotype was distinguished by nine SNPs. Plants were divided into two groups based on their height, and tall and short plants were distinguished and clustered according to the expression level of *OsPHq1*. QTLs and candidate genes related to plant height regulation, and thus, biomass regulation, were screened and identified in this study, but the molecular mechanism of the regulation remains poorly known. The information obtained in this study will help develop molecular markers for marker-assisted selection and breeding through rice plant height control.

## 1. Introduction

Rice provides food to more than half of the world’s population [1]. Asia represents more than 90% of the world’s rice production and is essential for the food security of these countries [2]. Rice also accounts for the calories of most African, South American, and Asian populations and is crucial for nutrition. The world population is rapidly increasing; therefore, improving rice yields is a central goal to feed everyone and solve the current food crisis [3].

Yield loss is increasing due to unpredictable and frequent environmental changes, and harvesting stable yields is essential and must remain sustainable for future agriculture [4]. The frequent occurrence of wars and the increasing frequency of pests and diseases seriously threaten food security [5]. Under these circumstances, providing a stable food supply to ensure nutrition has become the breeder’s main goal. Smart farms can prevent significant fluctuations in food supply due to the unpredictable climate and enable proactive responses [6]. The *sd1* gene, which led the Green Revolution by improving wheat self-sufficiency in 1970, interferes with gibberellin (GA) synthesis, improving yields significantly [7]. Climate change, the increasing frequency of pests and diseases, and the differentiation of new species threaten crops worldwide, and smart farms are being introduced to reduce environmentally caused problems [6]. Plant height must be adjusted for smart farms to breed cultivars suitable for plant factories, and such a strategy could lead to a second Green Revolution [8].

Rice yield includes biomass and yield index. Plant height is related to biomass [9] and is an essential agronomic trait involved in photosynthesis and lodging [10]. The Green Revolution led in 1970 reduced plant height and increased the yield index to increase rice yield [11]. However, the potential for yield index has been decreasing, and breeding strategies are accordingly aiming for biomass increase to improve rice yields. Therefore, rice breeders are working to improve plant height and select cultivars with a high yield potential. An extremely big plant can cause lodging and make machine harvesting more challenging, negatively affecting yield. Therefore, a cultivar with a desirable plant height is needed to allow for maximum breeding efficiency while minimizing negative impacts. Understanding the genetic pattern of plant height is key to establishing an efficient breeding strategy.

Important agronomic traits related to yield, such as plant height, panicle length, and tiller number, are inherited quantitatively and are subject to Mendel’s laws of inheritance [12]. Rice plant height is genetically controlled by quantitative trait loci (QTL) [13]. Research on QTL mapping has been systematically conducted on various crops [14,15]; it is one of the most efficient strategies for screening genes associated with key traits [16]. QTL mapping has provided a very useful approach to analyzing complex phenotypic traits, and QTLs related to the plant height of major food crops have also been identified [14,15]. Some have been cloned, and their gene functions have been characterized; major QTLs are widely applied in breeding programs [17,18]. Map-based cloning based on QTLs demonstrated the regulation of plant height by signals at the crosstalk of GAs, brassinosteroids, and cytokines [19,20]. Identifying QTL regions related to various traits in rice is essential for the physiological understanding of plant processes and increasing breeding efficiency.

Recently, *qSBM1* was identified in rice; it is involved in plant height increase and, ultimately, biomass increase by improving the spikelet number and yield [21]. However, rice is grown in paddy fields, and environmental requirements are essential for its growth. QTL mapping also detects the most likely related region involving environmental and genetic factors [22]. Because of its unpredictability, the environment can be a major obstacle to QTL mapping; therefore, detecting commonly identified regions by experimenting in various environments is essential [23]. Searching candidate genes by QTL mapping increases the speed of gene cloning and understanding gene function. Because the entire rice genome has been sequenced, it is possible to identify open reading frames (ORFs) in the QTL region of each chromosome. Rice plant height is an important agricultural characteristic that led to the Green Revolution. Therefore, it has been studied the most for a long time, and it is distributed on all 12 rice chromosomes, especially chromosomes 1, 3, and 4 (www.gramene.org/, accessed on 25 October 2022). Among these, *OsGA20ox1* [24], *OsGA20ox2* [25], *sd1* [26], and *IPA1* [27] were cloned and studied to show that their gene function strongly controls plant height.

In this study, the Cheongcheong/Nagdong double haploid (CNDH) population, derived from a cross between ‘Cheongcheong’ and ‘Nagdong’, was used for five years to identify QTL regions involved in plant height. In addition, candidate genes were screened in regions that were stably mapped, and homology and protein interactions were predicted. These results provide useful information for breeding rice cultivars with improved yield through increased plant height. Our study also suggests that the applied method can be used to efficiently identify genes regulating plant height.

## 2. Results

### 2.1. Phenotypic Variation in Plant Height

**Figure 1:**
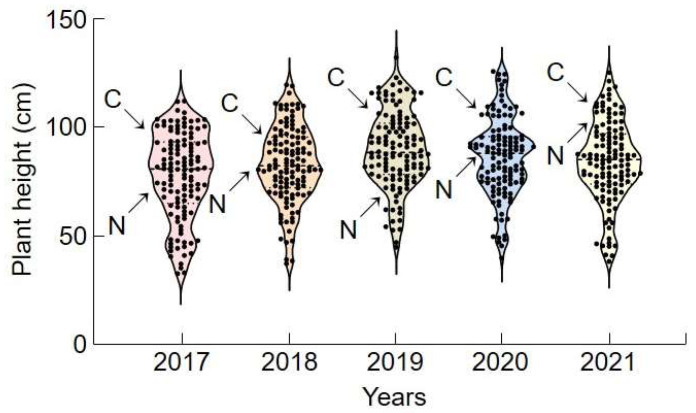
Violin plots of plant height frequency distribution for 120 CNDH individuals surveyed over five consecutive years. The plant heights of the CNDH population were normally distributed in all survey years, suggesting that plant height is a quantitative trait on which various genes can act. In all survey years, ‘Cheongcheong’ plants were taller than ‘Nagdong’ plants. The *x*-axis represents the year, and the *y*-axis represents the range of the plant height distribution of the CNDH population. C, ‘Cheongcheong’; N, ‘Nagdong’.

A QTL region was mapped by analyzing the height of ‘Cheongcheong’, ‘Nagdong’, and 120 CNDH populations over five years (2017–2021) (Figure 1). For those five years, ‘Cheongcheong’ plants were taller than ‘Nagdong’ plants, and the 120 CNDH-crossed individuals presented considerable variations. Cheongcheong plants were between 86.0 and 92.6 cm high, with an average of 89.0 ± 2.7 cm, while Nagdong plants were between 82.1 and 92.0 cm, with an average of 88.6 ± 4.6 cm. The plant height of the 120 CNDH individuals ranged between 32.7 and 129.2 cm, with an average of 82.7 ± 17.9 cm. Plant heights were continuous and normally distributed throughout the investigated period. These results indicate that plant height is suitable for QTL mapping. No significant difference was observed between the years, and the distribution frequency was similar. Broad-sense of heritability (h^2^) was calculated when each plant height was surveyed from 2017 to 2021. H^2^ was surveyed at 41.5% (2017), 46.8% (2018), 43.1% (2019), 44.3% (2020), and 48.1% (2021), and heritability was found to be less than 50% in all years surveyed. It was found that height is influenced more by environmental factors than by genetic control.

For the rest of the investigation, three lines with the tallest and shortest plants were selected for each cultivar. Among the 120 CNDH individuals, lines taller or shorter than individual ‘Cheongcheong’ or ‘Nagdong’ cultivars were selected. CNDH39-3, CNDH48-1, and CNDH79 were lines with the tallest plants for five consecutive years, and CNDH9, CNDH11, and CNDH45 were lines with the shortest plants for five consecutive years.

### 2.2. Identification of QTLs Related to Plant Height

**Figure 2:**
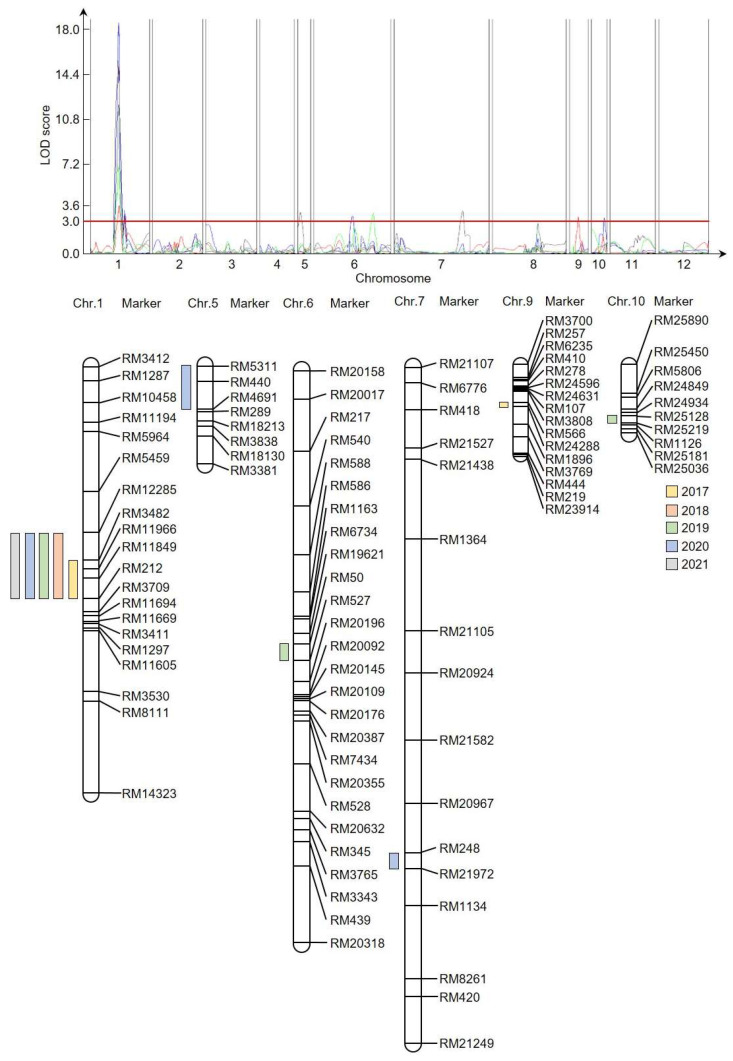
Genetic map of the CNDH population and QTL mapping related to plant height regulation. A total of 222 SSR markers were applied to the genetic map; they are evenly distributed across 12 chromosomes. QTLs were identified with a LOD score of 3.0 or higher on chromosomes 1, 5, 6, 7, 9, and 10. The RM3482-RM212 region on chromosome 1 was stably identified in the same region during the investigated period. The red line showing the LOD score is displayed to improve comprehension. Each color on the genetic map indicates the year in which the QTL was identified.

To identify QTL regions related to plant height, single-sequence repeat (SSR) markers were used to construct a genetic map for the CNDH population. To identify markers that could distinguish ‘Cheongcheong’ and ‘Nagdong’ populations by polymorphism, 788 SSRs were analyzed, and 423 were selected. Among those, 222 co-dominant SSRs displayed clear PCR bands, and a genetic map of the CNDH population was constructed based on those 222 selected SSRs. The 222 SSRs are evenly distributed across 12 chromosomes in rice, with 19–50 SSRs on each chromosome. The total length of the CNDH genetic population map is 2121.7 cM, and the average distance between SSRs is 10.6 cM (Figure 2). QTLs were detected by inclusive composite interval mapping using an empirical threshold LOD score of 3.0 or higher.

A genome-level analysis was conducted by applying the genetic map and plant height data collected from the 120 CNDH plants (Supplementary Table S2). The phenotypic variance explained (PVE) was 4.0–13.1%, and the LOD score was 2.7–17.6, with 13 various QTLs detected on chromosomes 1, 5, 6, 7, 9, and 11 (Figure 2). *qPh1* (LOD, 3.6; PVE, 10.8%) was identified in RM3482-RM212 on chromosome 1; *qPh1-1* (LOD, 11.4; PVE, 10.6%), *qPh1-3* (LOD, 17.6; PVE, 13.1%), *qPh1-5* (LOD, 14.7; PVE, 12.1%), and *qPh1-6* (LOD, 6.7; PVE, 9.3%) were identified in RM12285-RM212; and *qPh1-2* (LOD, 2.9; PVE, 4.8%) and *qPh1-4* (LOD, 3.1; PVE, 5.0%) were identified in RM3709-RM11669. *qPh5* (LOD, 3.2; PVE, 4.9%) was identified in RM5311-RM4691 on chromosome 5. *qPh6* (LOD, 2.8; PVE, 4.3%) and *qPh6-1* (LOD, 3.1; PVE, 6.9%) were identified in RM50-RM527 and RM20632-RM345 on chromosome 6, respectively. Finally, *qPh7* (LOD, 3.2; PVE, 5.4%), *qPh9* (LOD, 2.8; PVE, 7.5%), and *qPh10* (LOD, 2.7; PVE, 4.0%) were identified in RM248-RM21972 on chromosome 7, RM566-RM24288 on chromosome 9, and RM25128-RM25219 on chromosome 10, respectively. RM3482-RM212 on chromosome 1 was mapped in the same region for five consecutive years with a PVE of 9.3–13.1% and a LOD score of 3.6–17.6. *qPh1*, *qPh1-1*, *qPh1-3*, *qPh1-5*, and *qPh1-6* were identified on chromosome 1 in the same region for five years and were very stable, even in changing environments.

### 2.3. Candidate Genes Associated with Plant Height Based on QTL Mapping

**Figure 3:**
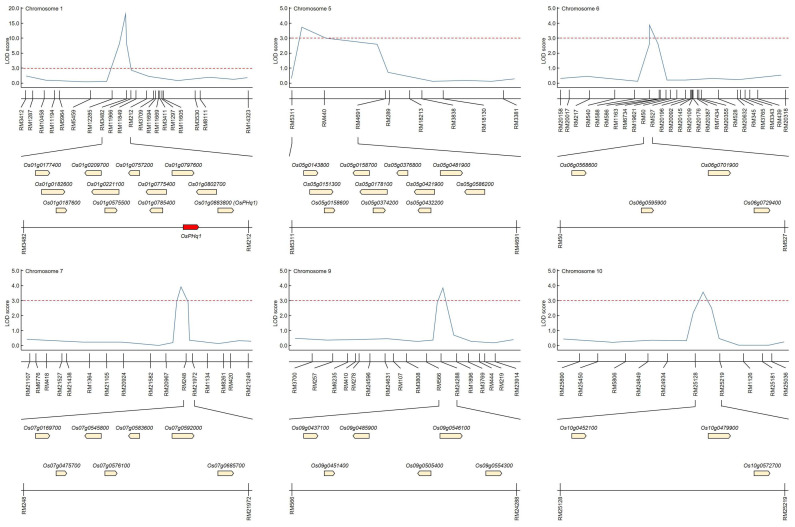
Physical map to screen potential candidate genes involved in plant height. Potential candidate genes involved in plant height regulation were screened in QTL regions identified with LOD scores of 3.0 or higher on chromosomes 1, 5, 6, 7, 9, and 10. Twelve potential candidate genes were screened in RM3482-RM212 and were stably mapped for five consecutive years. The red line indicates LOD score 3.0, and the blue line indicates the LOD score indicated by each marker.

The QTL mapping helped identify candidate genes that could potentially control plant height in the same SSR regions stably over the five study years. Candidate gene identification also included QTL regions identified only once during the five study years (Figure 3 and Supplementary Table S3). The RM11966-RM212 region on chromosome 1 has been mapped identically for five years, and RM3709-RM11669 was mapped in the same region on chromosome 1 for two consecutive years (2018–2019). RM5311-RM4691 on chromosome 5, RM20196-RM20092 and RM20632-RM345 on chromosome 6, RM248-RM21972 on chromosome 7, RM3808-RM566 on chromosome 9, and RM24934-RM25219 on chromosome 10 were identified only once in each region during the five years. The identified QTL regions were searched for potential candidate genes that may be involved in determining rice plant height. There were 12 in RM3482-RM212 on chromosome 1, 11 in RM5311-RM4691 on chromosome 5, 4 in RM50-RM527 on chromosome 6, 7 in RM248-RM21972 on chromosome 7, and RM25128- on chromosome 10. In RM25219, three potential candidate genes were screened.

GA 3beta-hydroxylase (*Os01g0177400*), GIGANTEA protein (*Os01g0182600*), cytokinin dehydrogenase 1 precursor-like (*Os01g0187600*), GA 2-oxidase 5-like (*Os01g0209700*), GH3 auxin-responsive promoter family protein (*Os01g0221100*), stomatal cytokinesis defective-like (*Os01g0575500*), GA 2-oxidase 4-like (*Os01g0757200*), cytokinin dehydrogenase 5 precursor-like (*Os01g0775400*), GH3 auxin-responsive promoter family protein (*Os01g0785400*), ethylene-responsive element binding factor3-like (*Os01g0797600*), auxin efflux carrier family protein (*Os01g0802700*), and GA 20 oxidase 2 (*Os01g0883800*) were screened in RM11966-RM212 on chromosome 1. In RM5311-RM4691 on chromosome 5, GH3 auxin-responsive promoter family protein (*Os05g0143800*), rubber elongation factor family protein (*Os05g0151300*), OsGA2ox1 (*Os05g0158600*), OsGA2ox1-like (*Os05g0158700*), GA 3beta-hydroxylase-like (*Os05g0178100*), cytokinin dehydrogenase 1 precursor-like (*Os05g0374200*), GA-regulated protein family protein (*Os05g0376800*), GA 20 oxidase 2-like (*Os05g0421900*), GA-regulated protein 2 precursor-like (*Os05g0432200*), auxin efflux carrier family protein (*Os05g0481900*), and GH3 auxin-responsive promoter family protein (*Os05g0586200*) were identified as being involved in plant height. Ent-kaurene oxidase 1-like (*Os06g0568600*), transcription elongation factor S-II (*Os06g0595900*), auxin-responsive SAUR protein family protein (*Os06g0701900*), and GA-regulated protein 2 precursor-like (*Os06g0729400*) were identified in RM20196-RM20092 and RM20632-RM345 on chromosome 6. GA 20-oxidase 3-like (*Os07g0169700*), auxin-responsive SAUR protein family protein (*Os07g0475700*), chitin-inducible GA-responsive protein (*Os07g0545800*), GH3 auxin-responsive promoter family protein (*Os07g0576100*), chitin-inducible GA-responsive protein (*Os07g0583600*), GA-regulated protein family protein (*Os07g0592000*), ethylene-insensitive 3 family protein (*Os07g0685700*) were identified in RM248-RM21972 on chromosome 7. Auxin-responsive SAUR protein family protein (*Os09g0437100*), 1-aminocyclopropane-1-carboxylate oxidase 1 (*Os09g0451400*), 60S ribosomal protein L9-like (*Os09g0485900*), auxin efflux carrier family protein (*Os09g0505400*), auxin-responsive SAUR protein family protein (*Os09g0546100*), and auxin efflux carrier family protein (*Os09g0554300*) were identified in RM3808-RM566 on chromosome 9. Finally, cytokinesis protein sepA-like (*Os10g0452100*), auxin response factor 10-like (*Os10g0479900*), and four-helical cytokine family protein (*Os10g0572700*) were identified in RM24934-RM25219 on chromosome 10.

### 2.4. Genetic Distance of Candidate Genes and Association Analysis

**Figure 4:**
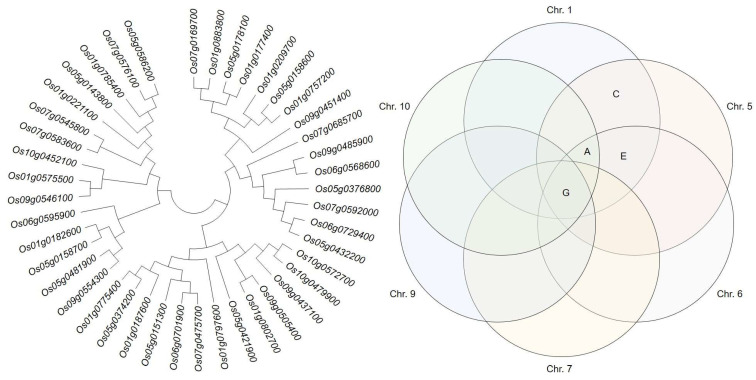
Screening and clustering of potential candidate genes involved in plant height regulation. A phylogenetic tree was constructed based on the genetic distance between candidate genes. Candidate genes were divided into three major groups and eight subgroups. They were screened from each chromosome and clustered by gene function; the result is presented as a Venn diagram. G, candidate gene involved in GA biosynthesis; A, candidate gene involved in auxin biosynthesis; E, candidate gene involved in ethylene biosynthesis; C, candidate gene involved in cytokinin biosynthesis.

Candidate genes identified from the selected QTL regions were grouped by genetic distance (Figure 4) and divided into three major and eight small groups. Major group 1 included 86.7% of GA-related and 13.3% of ethylene-related candidate genes. Major group 2 included 46.2% of candidate genes related to auxin synthesis and degradation and 30.8% of cytokine-related, 15.4% of GA-related, and 7.7% of ethylene-related candidate genes. Finally, the major group 3 included 53.3% of candidate genes related to auxin synthesis and degradation, 33.3% related to GA synthesis and degradation, and 13.3% related to cytokinin synthesis and degradation. All three major groups included candidate genes related to GA synthesis and degradation. However, candidate genes related to auxin and cytokinin synthesis and degradation were only present in groups 2 and 3, and candidate genes related to ethylene synthesis and degradation were only in groups 1 and 2.

### 2.5. Expression Level of Candidate Genes

**Figure 5:**
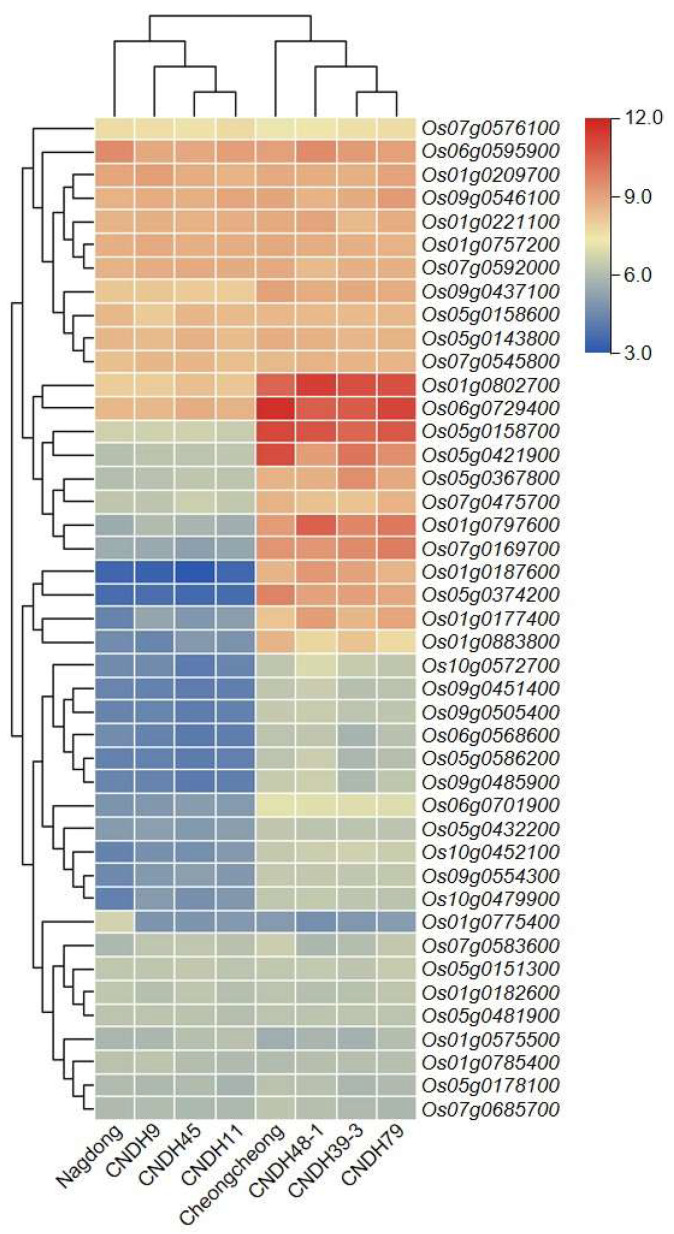
Heatmap presenting the expression level of potential candidate genes involved in plant height regulation. Potential candidate genes were divided into two major groups and four subgroups based on their expression level. Plants were divided into two groups based on their height. Taller plants (‘Cheongcheong’ CNDH39-3, CNDH48-1, and CNDH79) and shorter plants (‘Nagdong’, CNDH9, CNDH11, and CNDH45) were distinguished by the expression levels of candidate genes. *OsPHq1* expression level was higher in taller plants than in shorter ones.

The expression level of the abovementioned candidate genes was analyzed at the rice seedling stage (Figure 5) and visualized by a heat map. Based on their expression, plant lines were divided into two groups: tall plants (‘Cheongcheong’ CNDH39-3, CNDH48-1, and CNDH79) and short plants (‘Nagdong’ CNDH9, CNDH11, and CNDH45). Candidate genes were divided based on their expression level into two major groups and four subgroups. Major group 1 candidate genes were expressed above the medium level, and major group 2 candidate genes were expressed below the medium level. In other words, the expression level of group 1 genes was higher than that of group 2 genes. Regarding the four subgroups, subgroup 1 and 4 genes displayed similar expression levels between tall and short plants. However, the expression level of subgroup 1 genes was high, while that of subgroup 4 genes remained low. Subgroup 2 and 3 candidate genes were differentially expressed between tall and short plants. Subgroup 2 genes maintained a very strong expression level in tall plants and a medium expression level in short plants. Finally, subgroup 3 genes were highly expressed in tall plants and weakly expressed in short plants.

### 2.6. Haplotype Analysis of OsPHq1 Associated with Single-Nucleotide Polymorphism

**Figure 6:**
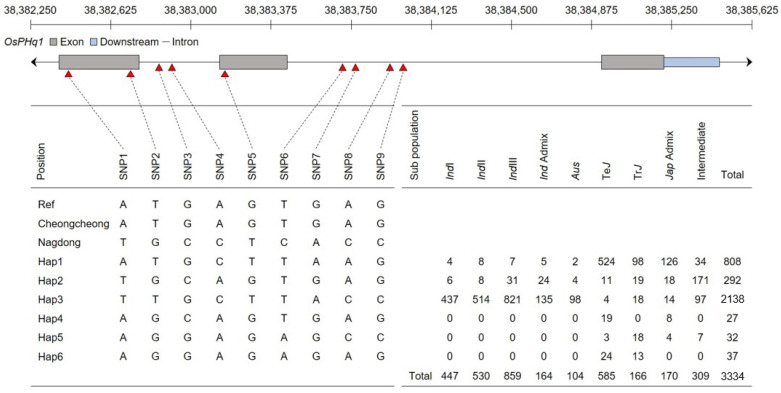
Haplotype analysis of *OsPHq1*. The schematic of *OsPHq1* represents the exon, the intron, and the downstream regions. *OsPHq1* was divided into six haplotypes based on nine different SNPs. Three SNPs were identified in the exon, six in the intron, and none in the upstream and downstream regions. Most subpopulations can be described as Hap4, which is distributed in the *Indica* group.

The rice single-nucleotide polymorphism (SNP) database was used for the haplotype analysis of *OsPHq1*. No SNPs were detected upstream or downstream of *OsPHq1*. However, three SNPs were detected in the *OsPHq1*exon and six in the intron (Figure 6): SNP1 (A > T), SNP2 (G > T), SNP3 (G > C), SNP4 (A > C), SNP5 (G > T), SNP6 (T > C), SNP7 (G > A), SNP8 (A > C), and SNP9 (G > C). Based on the combination of detected SNPs, six haplotypes were identified. Using the Rice Genome Annotation Project Database, the highest ratio was found for *hap4* and confirmed in the ‘*indica*’ group.

### 2.7. OsPHq1 DNA and Protein Sequence Analysis

**Figure 7:**
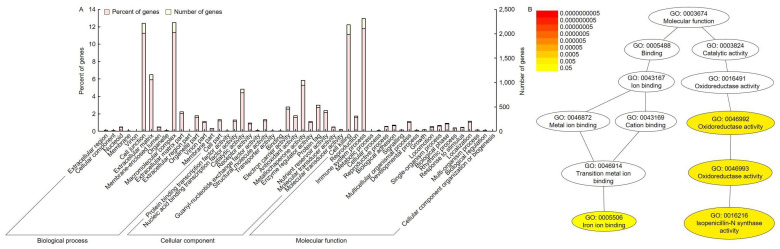
Ontology analysis and pathway annotation of potential candidate genes involved in plant height regulation. (**A**) GO terms were divided into three categories: biological process, cellular component, and molecular function. (**B**) Additionally, pathways expected to be differentially expressed in potential candidate genes were classified. The 12 most enriched pathways are indicated. The gradient color of each cluster included in the network represents the *p*-value of each cluster.

**Figure 8:**
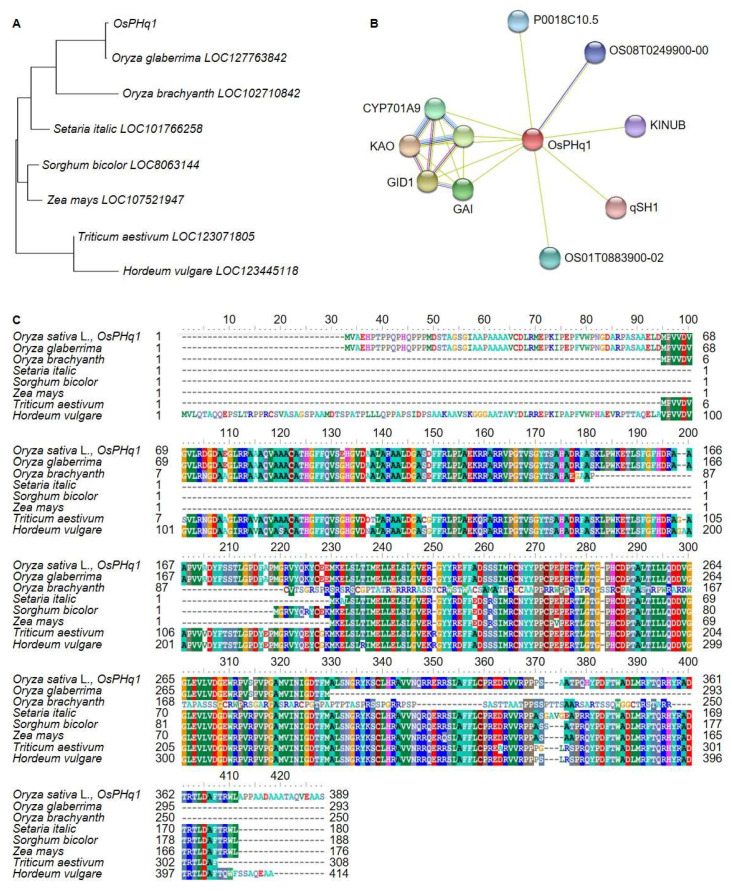
Genetic distance and homology analysis of *OsPHq1*. *OsPHq1* is similar in DNA and protein sequence to the GA 20 oxidase 2 of *O. brachyantha*, *O. glaberrima*, *S. italic*, *S. bicolor*, *Z. mays*, *T. aestivum*, and *H. vulgare*, and has a very close genetic distance to *O. glaberrima*. (**A**) *OsPHq1* is very similar to the DNA sequence of O. *glaberrima*, and the genetic distance is close. (**B**) In addition, *OsPHq1* is involved in the control of plant height by interacting with proteins related to hormone signaling. (**C**) The protein sequence of *OsPHq1* is homologous to GA 20 oxidase 2. The colors shown in each figure indicate that they perform the same function. It makes it easier to visually recognize the same thing.

*OsPHq1* DNA and protein sequences were analyzed. DNA analysis was conducted using the BLAST function of NCBI. The *OsPHq1* screened from the RM22861-RM6999 on chromosome 1 encodes a GA 20 oxidase 2 (semidwarf-1) protein (Figure 7). A phylogenetic tree was built based on the genetic distance between Gramineae GA 20 oxidase 2 DNA sequences (Figure 8). The tree indicated that the *OsPHq1* group was genetically close to *O. glaberrima* (identity 99.4%, similarity 84.0%) and *O. brachyantha* (93.2% identity, 92.0% similarity) GA 20 oxidase 2, while *Triticum aestivum* (88.5% identity, 87.0% similarity) and *Hordeum vulgare* (88.2% identity, 86.0% similarity) displayed the greatest genetic distance from the *OsPHq1* group. The *Os*PHQ1 protein sequence analysis indicated homology with the *O. brachyantha*, *O. glaberrima*, *Setaria italic*, *Sorghum bicolor*, *Zea mays*, *T. aestivum*, and *H. vulgare* GA 20 oxidase 2 protein sequences. *Os*PHQ1 controls rice plant height by interacting with several factors involved in GA biosynthesis and signaling: GID1, which interacts with the DELLA protein SLR1, a transcriptional factor repressing GA signaling; KAO, involved in GA biosynthesis; A0A0P0WS15, a putative acyl-activating enzyme 19; 1-aminocyclopropane-1-carboxylate synthase; OSH15, a putative transcription factor that might be involved in shoot formation during embryogenesis; qSH1, a putative transcription factor; A0A0P0XZM3, autophagy-related protein 13b; Q6H657_ORYSJ, autophagy-related protein 13a; and Q0JBE1_ORYSJ, cDNA clone:J023108N03.

## 3. Discussion

Plant height is an important agronomic trait closely related to lodging resistance, biomass, yield, and mechanized harvest in rice and most crops [9,28]. The *sd1* and *Rht-B1*/*Rht D1* genes, involved in the control of plant height in rice, were previously cloned and characterized and successfully applied to breed semidwarf cultivars with reduced plant height. Ultimately, *sd1* and *Rht-B1*/*Rht D1* served as keys for the “green revolution” [29,30,31]. In addition, *SPS* was identified through QTL mapping and effectively applied to increase biomass by controlling plant height [32]. Because rice plant height is greatly influenced by the environment, studying plants in various environments and identifying stable QTL regions is essential [13]. Marker-assisted breeding has the potential to achieve a high genetic advantage in a shorter time by selecting QTLs and linkage markers for target traits and provides high efficiency for trait selection and positive QTL introduction [33]. Several studies were conducted to identify QTLs involved in rice plant height control. *LOC_Os12g40890* (*qPH12*) is a candidate gene related to plant height regulation screened through QTL mapping in NIL lines derived from a backcross [34]. *LOC_Os12g40890* was identified in the Indel12-29 and Indel12-31 regions and was reported to be involved in plant height depending on sequence insertion or deletion. In addition, various haplotypes searched in the *OsPH9* sequence were detected through QTL mapping and were shown to determine plant height [35]. Most of the previously reported candidate genes are related to plant hormones, such as GA and auxin, which are related to plant height control [36]. In fact, when these hormones act positively, cell length increases, ultimately increasing plant height [37]. However, the mapped QTLs are distributed on various chromosomes in each study, and selecting QTLs that can be effectively applied is very challenging. One explanation is that the mapping population used in each study and the environment are different [22,38]. Therefore, identifying QTLs that are consistently stable for successive years and under various environments is essential to determine those involved in rice plant height control [39].

In this study, a rice QTL involved in plant height control was identified consistently for five consecutive years (from 2017) using a double haploid population derived by another culturing F_1_ from a ‘Cheongcheong’ × ‘Nagdong’ crossing and surveying their height. Among the identified QTLs, RM3482-RM212 was detected in the same region for five consecutive years. The stably mapped QTL provided an opportunity to carefully screen potential candidate genes involved in plant height control, even in changing environments [40,41]. The QTL mapped in this study can be used to develop molecular markers to support marker-assisted selection.

Thirteen potential candidate genes involved in rice plant height control were screened in RM3482-RM212. A total of 8, 5, 3, 9, 10, 2, and 5 potential candidate genes associated with plant height were screened from RM2343-RM52342, RM2343-RM52342, RM2343-RM52342, RM2343-RM52342, RM2343-RM52342, RM2343-RM52342, and RM2343-RM52342, respectively. However, because rice plant height is determined by the complex interaction of numerous genes, selecting candidate genes using only the rice database description presents limits. The expression level of the screened candidate genes was analyzed at the rice seedling stage and clustered accordingly. The rice genome is similar to the genome of other Graminae crops; as a result, the gene homology analysis indicated the homology of *OsPHq1* to the sequence of GA 20 oxidase 2 in Graminae. The screened candidate genes were applied to the CNDH population, and their expression levels were analyzed. The CNDH population has already had advanced generations in rice fields for 10 years, and its agricultural traits have been fixed. And, among these, segregation lines were eliminated. The CNDH population was previously applied to analyze the expression levels of various genes [42,43]. However, in order to fully understand gene function, it is necessary to develop a genome-editing plant or transgenic plant using CRISPR/Cas9.

GA acts to increase cell length in rice, ultimately increasing plant height [44]. GA 20 oxidase 2 has been cloned from rice, maize, and wheat and is involved in plant height control [24,45,46]. It interferes with GA activity and induces a dwarf phenotype [24]. *OsPHq1* is not completely identical to the previously reported GA 20 oxidase 2 of *O. sativa* japonica and indica groups but is genetically very close. Nine SNPs were identified in the DNA sequence of *OsPHq1* and were differentially expressed between short and tall plants. *OsPHq1* expression level is stronger in short plants than in tall ones, suggesting its role in dwarfism. However, additional research is needed to clearly understand *OsPHq1* function and develop transgenic or genome-edited plants.

In this study, *OsPHq1*, a candidate gene potentially involved in rice plant height regulation, was identified. Plant height is closely related to biomass production; therefore, selection efficiency could be increased using *OsPHq1* as a molecular marker to breed crops with increased biomass through plant height control.

## 4. Materials and Methods

### 4.1. Plant Materials and Field Trials

The 120 CNDH populations derived by crossing ‘Cheongcheong’ and ‘Nagdong’ were used to map QTLs related to plant height regulation. ‘Cheongcheong’ and ‘Nagdong’ plants were obtained from Prof. Kyung-Min Kim of the Kyungpook National University Plant Molecular Breeding Laboratory in Gunwi, Republic of Korea. The 120 CNDH individuals were bred and cultivated for five consecutive years (2017–2021). The field tests were repeated three times each year in a completely randomized field design. In this study, all plants were cultivated in compliance with international guidelines and legislation provided by the RDA. Additionally, local practices were applied for breeding and cultivation, and the Convention on the Trade in Endangered Species of Wild Fauna and Flora (https://www.cites.org/, accessed on 10 April 2017) was respected. The seeds were sterilized with a disinfectant solution (HANKOOKSAMGONG, Cat. 1502-7, Seoul, Republic of Korea) under darkness at 25 °C for 4 days. Sterilized seeds were placed in 50 trays on 20 April 2017, 21 April 2018, 23 April 2019, 25 April 2020, and 24 April 2021. Thirty days after sowing, each line was transplanted to six rows in the field. Twenty-five plants were included per row, and the planting distance was 30 × 15 cm. During the rice growth period, (N-P_2_O_5_-K_2_O, 9-4.5-5.7 kg/10a) was applied as a fertilizer. Nitrogen was applied in stages of basal:tillering:panicle, at a ratio of 5:2:3. Potassium was also divided into basal:panicle, with a ratio of 7:3. The entire amount of phosphoric acid was applied basally. All cultivation management methods complied with the standards presented by the RDA.

Every year, the height of ‘Cheonghcheong’, ‘Nagdong’, and CNDH plants was investigated in the field starting from 40 days after transfer in the field. Plant height was recorded by measuring the length of the aboveground main stem from its base [9]. Ten plants were randomly selected from each population and examined. For the plant height surveyed each year, a correlation coefficient was calculated to construct a heat map. Clustering was performed using the correlation coefficient, and the ‘Cheongcheong’, ‘Nagdong’, and CNDH plants were clustered into groups according to their plant height surveyed for five years.

### 4.2. DNA Extraction and PCR for Genetic Map

Leaf samples from ‘Cheongcheong’, ‘Nagdong’, and CNDH populations were ground using Tissue Lyser II (QIAGEN, Hilden, Germany). Genomic DNA was extracted using the NucleoSpin Plant II Kit (Macherey–Nagel GmbH & Co., KG, Deutsch, Düren, Germany). A total of 10 μL of RNase and 300 μL of PL2 buffer were added to 150 mg of ground leaf samples. Samples were thoroughly mixed using a vortex and incubated at 65 °C for 30 min. Then, 75 μL of PL3 buffer was added, and samples were incubated at 4 °C for 10 min. Centrifugation was performed at 13,000 rpm for 1 min, the supernatant was retrieved using the NucleoSpin Filter (violet ring) provided in the kit, and samples were centrifuged again at 13,000 rpm at 25 °C for 10 min. The filtered solution was transferred to a new 1.5 mL Eppendorf tube, and 450 μL of PC buffer was immediately added and carefully mixed by pipetting. The mixed solution was transferred to the NucleoSpin Plant II Column (green ring) provided in the kit, and samples were centrifuged at 25 °C at 13,000 RPM for 3 min. The filtered solution was discarded, 400 µL of PW1 buffer was added, and samples were centrifuged for 1 min. Then, 200 µL of PW2 buffer was added, and samples were centrifuged for 2 min. In each step, all solutions filtered after centrifugation were discarded. After washing with PW2, the NucleoSpin Plant II Column (green ring) was transferred to a new 1.5 mL Eppendorf tube, 50 μL of PE buffer was added, and samples were incubated at 65 °C for 10 min. Finally, samples were centrifuged at 13,000 rpm for 1 min. After centrifugation, the DNA was collected in a 1.5 mL Eppendorf tube, and samples were stored at −20 °C. The extracted genomic DNA was diluted to 100 ng/μL using NanoDrop 2000 (ThermoFisher Scientific, Cat. ND2000USCAN, Seoul, Republic of Korea) and checked for quality by electrophoresis on a 0.8% agarose gel. A PCR mixture was prepared for amplification, including 100 ng of template DNA, 2.5 mM deoxy-nucleo-triphosphates, 10× buffer (50 mM KCl, 20 mM Tris-HCl (pH 8.0), 2.0 mM MgCl_2_), 20 pmol forward primer, 20 pmol reverse primer, 1 unit, and HS Prime Taq polymerase (cat. no. G-7002; Genet Bio, Daejeon, Republic of Korea). Distilled water was added for a total volume of 50 μL. Samples were amplified by PCR (Bio-Rad, Hercules, CA, USA) with the following thermocycle: predenaturation at 95 °C for 5 min; 35 cycles of denaturation (95 °C, 30 s), annealing (55 °C, 30 s), and extension (72 °C, 30 s); and a final extension at 72 °C for 5 min to ensure sufficient amplification of the gene fragment. PCR products were stored at 4 °C.

### 4.3. QTL Mapping and Meta-Analysis

The genetic mapping of the CNDH population was performed using Mapmaker version 3.0 [47] by applying 222 SSR markers. The average distance between markers was 10.6 cM. QTLs related to rice plant height regulation were analyzed by applying the composite interval mapping function of WinQTLCart 2.5 software [48] with a threshold value of LOD ≥ 3.0. A 1000-permutation test was performed [49]. The PVE (%) and additive effects according to 1 cM of the genetic map were analyzed at a 95% confidence level. The confidence interval was defined as the LOD reduction area around the LOD score of the detected QTL peaks. The identified QTLs were nomenclatured according to the method suggested by McCouch [50]. QTLs identified in different environments but mapped in the same region were estimated through meta-analysis of consistent QTLs using BioMercator 2.1 software [51].

### 4.4. Screening of Candidate Genes in QTL Regions

Various programs related to gene information were used to screen candidate genes related to plant height regulation. The SSR names of the detected QTL regions were searched on Rapdb (https://rapdb.dna.affrc.go.jp/, accessed on 8 October 2021) and RiceXpro (https://ricexpro.dna.affrc.go.jp/, accessed on 8 October 2021), and information about various ORFs in the marker interval was obtained. The searched ORFs clustered according to various functions, and candidate genes were identified using the plant height filter. The phylogenetic trees of the candidate genes were constructed using MEGA 11.0 (https://www.megasoftware.net/, accessed on 13 October 2021) based on genetic distance. The candidate genes were screened among Graminae crop sequences using NCBI (http://www.ncbi.nim.nih.gov, accessed on 21 October 2021) and BioEdit 7.0 (www.mbio.ncsu.edu/BioEdit/BioEdit.html, accessed on 21 October 2021). BLAST was used for homology analysis, and protein interactions were predicted using ExPASy (https://www.expasy.org, accessed on 25 October 2021) and Simple Modular Architecture Research Tool (http://smart.embl-heidelberg.de/, accessed on 25 October 2021).

### 4.5. RNA Extraction and Expression Level Analysis

RNA was extracted from CNDH short and tall individuals. To improve RNA quality and quantity, all the experimental equipment was washed with DEPC. RNA was extracted from each line sample following the manual provided by the RNeasy plant mini kit (QIAGEN, Germany). In the final step, RNA was eluded into 50 µL of RNase-free water. The RNA extract concentration was analyzed by the ultramicrospectrophotometer ND-2000 (Thermo Fisher Scientific, MA, USA). A total of 1 μg of extracted RNA was used for cDNA synthesis using the qPCRBIO cDNA Synthesis Kit (PCRBIOSYSTEMS, London, UK). According to the provided manual, 4 µL of 5× cDNA synthesis mix and 1 µL of 20× RTase were added for a final volume of 20 μL adjusted with RNase-free water. The Eco Real-Time PCR System was used with 1 μL of cDNA as a template. The qPCR mixture included 10 µL of 2× qRCRBIO SyGreen Blue Mix, 1 µL of cDNA, 0.5 µL of forward primer (20 pmol/µL), and 0.5 µL of reverse primer (20 pmol/µL), for a total volume of 20 µL adjusted with ddH_2_O. *OsActin* was used as a control, and each reaction was performed in triplicate. The means and standard deviations were analyzed (Supplementary Table S1).

### 4.6. Statistical Analysis

R (version 4.1.3, The R Foundation for Statistical Computing) was used for statistical analyses. The characteristics of each line were randomly examined in ten plants and in triplicate. The agricolate package was applied to analyze the average of the investigated characteristics, and Duncan’s multiple range test (DMRT) was used to compare the averages. Additionally, the significance of the average value was analyzed with *p* < 0.05 according to the Student’s *t*-test and DMRT. Pearson’s correlation coefficient was used with the psych package to clearly analyze the relationship between the investigated traits each year. Finally, corr.test was applied to construct a heatmap, and clusters were created based on the obtained Pearson’s correlation coefficient.

## 5. Conclusions

Rice plant height is a trait significantly related to yield and biomass. Fourteen potential candidate genes related to plant height regulation, including *OsPHq1*, were screened from the RM3482-RM212 on chromosome 1. Genetic QTL mapping was conducted for five years, and *OsPHq1* was identified as encoding a GA 20 oxidase 2-like protein, and its DNA and protein sequences share homology with other Graminae crops. *OsPHq1* clusters were differentially expressed depending on whether the plant belonged to the taller group or the shorter one. The haplotype analysis identified nine SNPs potentially playing a role in plant height control. In the future, we plan to investigate the molecular mechanism of *OsPHq1* function by breeding genome-edited lines using CRISPR/Cas9.

## Data Availability

Upon request to the corresponding author of this article.

## Supplementary Materials

The following supporting information can be downloaded at: https://www.mdpi.com/article/10.3390/ijms242316895/s1.
