# Supplementary material for: Quantitative Trait Loci Mapping Identified Candidate Genes Involved in Plant Height Regulation in Rice
Source: Int J Mol Sci. 2023 Nov 29;24(23):16895. doi: 10.3390/ijms242316895 (PMC10706376; doi:10.3390/ijms242316895)
Supplement: Supplementary file 1 [file ijms-24-16895-s001.zip › ijms-2724290-supplementary/IJMS_supporting information.docx]

**SUPPORTING INFORMATION**

**Table S1.** qRT-PCR primer set for analyzing expression levels of candidate genes.

| Primer name | Forward/Reverse | Sequence (5’ to 3’) |
| --- | --- | --- |
| *OsActin* | Forward | ACCACAGGTATTGTGTTGGACTC |
|  | Reverse | AGAGCATATCCTTCATAGATGGG |
| *Os01g0177400* | Forward | ACCTCCTCTTCTGTGACGTG |
|  | Reverse | CCCTCAAGAACAACCTCAGC |
| *Os01g0182600* | Forward | CTCATCGTCGAATGGGGAGA |
|  | Reverse | ATGGTGGTTGATAGACGGCA |
| *Os01g0187600* | Forward | AGGAGGTGTTCTACGTGGTG |
|  | Reverse | AGTATCCTCCTGTTCTGCGC |
| *Os01g0209700* | Forward | CCCTCCAGGTGCTAACGAA |
|  | Reverse | ATGGACACCCTCGACTTCT |
| *Os01g0221100* | Forward | CATCTACAGCAGCAGGGAGA |
|  | Reverse | CTCATCGTCGCCTTGAACTC |
| *Os01g0575500* | Forward | AAAGGACAACAACCGCCATC |
|  | Reverse | GCACCCAATTTCGACCCAAT |
| *Os01g0757200* | Forward | TCAGGTTCTTCTCCCAGACG |
|  | Reverse | CATTGAACCCGATCCGCTTG |
| *Os01g0775400* | Forward | TACCCCATGAACAGGCACAA |
|  | Reverse | CACCAGGTAGAACACGTCCT |
| *Os01g0785400* | Forward | CTACTTCGGCCTCAACCTGA |
|  | Reverse | TTGTTGCAATTGACGGGGAG |
| *Os01g0797600* | Forward | CCGTTTGCATCACAGTCGAT |
|  | Reverse | AATCCAAGTCCAGAGTCGGC |
| *Os01g0802700* | Forward | GTTCACCATTCGTACCACCG |
|  | Reverse | CGCTATCTTGTATCCGCACG |
| *Os01g0883800* | Forward | ACTGCGAGGAGATGAAGGAG |
|  | Reverse | CGCGAAGAACTCCCTGTAGT |
| *Os05g0143800* | Forward | GTTCATCCCGTTCGACATGG |
|  | Reverse | CACCAACTCGTACGTCTTGC |
| *Os05g0151300* | Forward | GAGGAGGTGACGGTGGAGAA |
|  | Reverse | CCAGATACCTCAGCCTCTCC |
| *Os05g0158600* | Forward | AGGCTAGCAATGGTGGGAAT |
|  | Reverse | TGTAGCCCTTCCACATCGTT |
| *Os05g0158700* | Forward | TATGCTCTTTAGGGGCCTGG |
|  | Reverse | TCAAATGCAACAGACAGGGG |
| *Os05g0178100* | Forward | GCGATCTCTTCCATGTGCTC |
|  | Reverse | CGAGGAAGTAGGGCATGGAG |
| *Os05g0374200* | Forward | GTTACTTGCCCTTGGTGGTG |
|  | Reverse | ATCGTGGAAGCTGAAATGGC |
| *Os05g0376800* | Forward | ACCGCCTTCCCCATGTAC |
|  | Reverse | CAGAAGAACATGCACGGCTT |
| *Os05g0421900* | Forward | CCTTCGGTTTCAATGGGAGC |
|  | Reverse | GGTCCATGCTGTGTAGAGGA |
| *Os05g0432200* | Forward | CAGTATGGCCAAGTCGCTCT |
|  | Reverse | ATGTGGAGCAGTAGTCTCGA |
| *Os05g0481900* | Forward | AGTGGCATTGATTGTACGGC |
|  | Reverse | ATTGGTCGCGACATAATGCC |
| *Os05g0586200* | Forward | GTACCTGCAGAACTTTGGGC |
|  | Reverse | CCGCTGGATATAGGGCTCAA |
| *Os06g0568600* | Forward | ACACAGCTGACAGATCACCA |
|  | Reverse | GTTTGTCAGGGTTCTTGGCA |
| *Os06g0595900* | Forward | GAGCAGCTTAACCGTGACAG |
|  | Reverse | GGTTTCTGGAGCCATCGTTC |
| *Os06g0701900* | Forward | GATCATCAGGGTGGCGGT |
|  | Reverse | CTTCCACCTCTTGAGCAGCT |
| *Os06g0729400* | Forward | ATGGCTTCTTCGTCCCTTCT |
|  | Reverse | AGAAGCATCGACATGGAGGA |
| *Os07g0169700* | Forward | TCACCTTCTTCCTCAACCCG |
|  | Reverse | CTCTGCGTGAACTCGAGGAA |
| *Os07g0475700* | Forward | ATCGTGCTCCCTTGCTCC |
|  | Reverse | CGGCGAGCACTTGTTCTTG |
| *Os07g0545800* | Forward | TCGTGGTGAAGGTCTTGACA |
|  | Reverse | CTTTCGTGACTTGTGTGGCA |
| *Os07g0576100* | Forward | TATGCTTCCTCCGAGTGCTT |
|  | Reverse | TTGGGCATGATGGTGTAGGA |
| *Os07g0583600* | Forward | AGGCTGTCAAGGGAGAAGAC |
|  | Reverse | GAGAGAGATCCACTGAGCCC |
| *Os07g0592000* | Forward | ATCTCACCGTCGCTGCAG |
|  | Reverse | CATCTTGCACAGCCCCATG |
| *Os07g0685700* | Forward | GGGAACAACAACCTGCAGAA |
|  | Reverse | CTTCCTTCATCAGCGGCATG |
| *Os09g0437100* | Forward | GCCACCCGATGTTCCAGAA |
|  | Reverse | CGTTCTCCAGCATCCAGAGG |
| *Os09g0451400* | Forward | CAAGGACCACTACAAGCGTG |
|  | Reverse | AAGAAGGTGCTCTCCCAGTC |
| *Os09g0485900* | Forward | AGAAGGTGAAGGACGAGCTC |
|  | Reverse | CACGTGGCATTTCTGGTTGA |
| *Os09g0505400* | Forward | AACAGGTGGCATGTCGAAAC |
|  | Reverse | CTGCAACGCCATGAACAAAC |
| *Os09g0546100* | Forward | AAGGGCCACTGTGTCATGTA |
|  | Reverse | CCATCGCTCGTGAAACCAAA |
| *Os09g0554300* | Forward | GGATTCTTGCCACACGATCC |
|  | Reverse | CAAACAGCTGAGCCATGGTT |
| *Os10g0452100* | Forward | GAGGTGGTGGTGGGTCAAA |
|  | Reverse | TCCGTCTCCACTAGCATACG |
| *Os10g0479900* | Forward | GTCCTAGCAACAGCCCACTA |
|  | Reverse | ACCAAATTGAGCATGCCTGG |
| *Os10g0572700* | Forward | CAAGGTTGGAAGAGGTGCAC |
|  | Reverse | CCATGGGAACGTCTAGGAGG |

**Table S2.** QTL information for plant height identified in the Cheongcheong/Nagdong double haploid population.

| Characteristics | Year | QTL | Chromosome | Marker interval^z^ | LOD | Additive effect^y^ | *R^2^*^x^ | Increasing effects^w^ |
| --- | --- | --- | --- | --- | --- | --- | --- | --- |
| Plant height (cm) | 2017 | qPh1 | 1 | RM3482-RM212 | 3.6 | -10.8 | 0.1 | Nagdong |
|  |  | qPh9 | 9 | RM566-RM24288 | 2.8 | 7.5 | 0.1 | Cheongcheong |
|  | 2018 | qPh1-1 | 1 | RM12285-RM212 | 11.4 | -10.6 | 0.3 | Nagdong |
|  |  | qPh1-2 | 1 | RM3709-RM11669 | 2.9 | 4.8 | 0.1 | Cheongcheong |
|  | 2018 | qPh1-3 | 1 | RM12285-RM212 | 17.6 | 13.1 | 0.4 | Cheongcheong |
|  |  | qPh1-4 | 1 | RM3709-RM11669 | 3.1 | -5.0 | 0.1 | Nagdong |
|  |  | qPh6 | 6 | RM50-RM527 | 2.8 | 4.3 | 0.1 | Cheongcheong |
|  |  | qPh10 | 10 | RM25128-RM25219 | 2.7 | -4.0 | 0.1 | Nagdong |
|  | 2020 | qPh1-5 | 1 | RM12285-RM212 | 14.7 | 12.1 | 0.4 | Cheongcheong |
|  |  | qPh5 | 5 | RM5311-RM4691 | 3.2 | 4.9 | 0.1 | Cheongcheong |
|  |  | qPh7 | 7 | RM248-RM21972 | 3.2 | 5.4 | 0.1 | Cheongcheong |
|  | 2021 | qPh1-6 | 1 | RM12285-RM212 | 6.7 | 9.3 | 0.2 | Cheongcheong |
|  |  | qPh6-1 | 6 | RM20632-RM345 | 3.1 | 6.9 | 0.1 | Cheongcheong |

^z^ Interval markers are those within the significance threshold on each border of the QTL range

^y^ Positive values of the additive effect indicate that alleles from Cheongcheong are in the direction of increasing the traits

^x^ The proportion of evaluated phenotype variation attributable to a particular QTL was estimated by the coefficient of determination (*R^2^*)

^w^ Increase allele is the source of the allele causing an increase in the measured trait

**Table S3.** Information of potential candidate genes.

| Chromosome | Marker interval | Locus | Description |
| --- | --- | --- | --- |
| 1 | RM3482-RM212 | *Os01g0177400* | GA 3beta-hydroxylase. |
|  |  | *Os01g0182600* | GIGANTEA protein. |
|  |  | *Os01g0187600* | Similar to Cytokinin dehydrogenase 1 precursor (EC 1.5.99.12) |
|  |  | *Os01g0209700* | Similar to GA 2-oxidase 5. |
|  |  | *Os01g0221100* | GH3 auxin-responsive promoter family protein. |
|  |  | *Os01g0575500* | Similar to Stomatal cytokinesis defective. |
|  |  | *Os01g0757200* | Similar to GA 2-oxidase 4. |
|  |  | *Os01g0775400* | Similar to Cytokinin dehydrogenase 5 precursor (EC 1.5.99.12) |
|  |  | *Os01g0785400* | GH3 auxin-responsive promoter family protein. |
|  |  | *Os01g0797600* | Ethylene responsive element binding factor3 (OsERF3). |
|  |  | *Os01g0802700* | Auxin Efflux Carrier family protein. |
|  |  | *Os01g0883800* | Gibberellin 20 oxidase 2 (EC 1.14.11.-) (Gibberellin C-20 oxidase 2) |
| 5 | RM5311-RM4691 | *Os05g0143800* | GH3 auxin-responsive promoter family protein. |
|  |  | *Os05g0151300* | Rubber elongation factor family protein. |
|  |  | *Os05g0158600* | OsGA2ox1. |
|  |  | *Os05g0158700* | Similar to OsGA2ox1. |
|  |  | *Os05g0178100* | GA 3beta-hydroxylase. |
|  |  | *Os05g0374200* | Similar to Cytokinin dehydrogenase 1 precursor (EC 1.5.99.12) |
|  |  | *Os05g0376800* | Gibberellin regulated protein family protein. |
|  |  | *Os05g0421900* | Similar to Gibberellin 20 oxidase 2 (EC 1.14.11.-) |
|  |  | *Os05g0432200* | Similar to Gibberellin-regulated protein 2 precursor. |
|  |  | *Os05g0481900* | Auxin Efflux Carrier family protein. |
|  |  | *Os05g0586200* | GH3 auxin-responsive promoter family protein. |
| 6 | RM50-RM527 | *Os06g0568600* | Similar to Ent-kaurene oxidase 1 (Fragment). |
|  |  | *Os06g0595900* | Transcription elongation factor S-II, central region domain containing protein. |
|  |  | *Os06g0701900* | Auxin responsive SAUR protein family protein. |
|  |  | *Os06g0729400* | Similar to Gibberellin-regulated protein 2 precursor. |
| 7 | RM248-RM21972 | *Os07g0169700* | Similar to GA 20-oxidase 3. |
|  |  | *Os07g0475700* | Auxin responsive SAUR protein family protein. |
|  |  | *Os07g0545800* | Similar to Chitin-inducible gibberellin-responsive protein. |
|  |  | *Os07g0576100* | GH3 auxin-responsive promoter family protein. |
|  |  | *Os07g0583600* | Chitin-inducible gibberellin-responsive protein. |
|  |  | *Os07g0592000* | Gibberellin regulated protein family protein. |
|  |  | *Os07g0685700* | Ethylene insensitive 3 family protein. |
| 9 | RM566-RM24288 | *Os09g0437100* | Auxin responsive SAUR protein family protein. |
|  |  | *Os09g0451400* | 1-aminocyclopropane-1-carboxylate oxidase 1 (EC 1.14.17.4) (ACC oxidase 1) |
|  |  | *Os09g0485900* | Similar to 60S ribosomal protein L9 (Gibberellin-regulated protein GA). |
|  |  | *Os09g0505400* | Auxin Efflux Carrier family protein. |
|  |  | *Os09g0546100* | Auxin responsive SAUR protein family protein. |
|  |  | *Os09g0554300* | Auxin Efflux Carrier family protein. |
| 10 | RM25128-RM25219 | *Os10g0452100* | Similar to Cytokinesis protein sepA (FH1/2 protein) |
|  |  | *Os10g0479900* | Similar to Auxin response factor 10. |
|  |  | *Os10g0572700* | Four-helical cytokine family protein. |
